# Test–retest reliability of TMS motor evoked responses and silent periods during explosive voluntary isometric contractions

**DOI:** 10.1007/s00421-025-05707-3

**Published:** 2025-02-22

**Authors:** F. Castelli, O. S. Mian, A. Bruton, A. C. Valappil, N. A. Tillin

**Affiliations:** 1 University of Roehampton, School of Life and Health Sciences, Whitelands College, Holybourne Avenue, London, SW15 4JD UK; 2 Centre for Cognitive and Clinical Neuroscience, Sport Health and Exercise Sciences, Brunel University London, Uxbridge, UB8 3PH UK

**Keywords:** Corticospinal excitability, Transcranial magnetic stimulation, Explosive contractions, Test–retest, Rate of force development

## Abstract

**Purpose:**

This study assessed the test–retest reliability of TMS motor evoked potentials (MEPs) and silent periods at early, middle, and late phases of the rising time–torque curve during explosive voluntary contractions. We also investigated how the number of consecutively averaged measurements influenced reliability.

**Methods:**

On two separate occasions 3–7 days apart, 14 adults performed several isometric explosive (1-s) contractions of the knee extensors, some of which were superimposed with TMS to elicit MEPs in the superficial quadriceps. Of those with TMS, stimulation was timed to elicit MEPs at either 45 (early), 115 (middle), or 190 ms (late) following contraction onset (16 with-TMS contractions per time condition). TMS was also superimposed at the plateau of 15 separate MVCs. Test–retest intraclass correlation coefficient (ICC) and coefficient of variation (CV) were calculated for MEPs and silent periods consecutively averaged over 3 to 15 separate contractions.

**Results:**

No one condition/phase was more reliable than another. For MEP amplitude, in all conditions except the explosive late phase, ICCs generally increased, and CV decreased, with an increase in the number of averaged contractions, and were > 0.50 ICC and < 15% CV within seven contractions. For silent period, ICCs and CVs were unaffected by the number of consecutively averaged contractions and remained > 0.50 ICC and < 10% CV.

**Conclusion:**

Test–retest reliability of TMS responses is comparable between phases of explosive contraction and at the plateau of MVC. To maximise reliability of MEPs during explosive contractions or MVCs, we recommend future studies average data across more than the 3–5 contractions typically reported in the literature investigating MEPs at MVC plateau.

**Supplementary Information:**

The online version contains supplementary material available at 10.1007/s00421-025-05707-3.

## Introduction

The rate of torque development (RTD) measures the ability of muscles to rapidly increase force around a joint (Tillin and Folland 2014), and is functionally important where time to develop force is limited, such as sprinting (Tillin et al. 2013) or balance recovery (Sundstrup et al. 2010). RTD is often measured in early (0–50 ms), middle (50–100 ms) and late (> 100 ms) phases of explosive contraction performed from rest (Maffiuletti et al. 2016), and different physiological factors limit the RTD in these separate phases. One limiting factor is neural drive, which, when measured using via surface electromyography (EMG) amplitude or motor unit discharge rates, has been shown to positively correlate with early- and middle-phase RTD (Folland et al. 2014; Del Vecchio et al. 2019). Despite the relevance of neural drive to RTD, the corticospinal mechanisms affecting RTD are not well established.

Transcranial magnetic stimulation (TMS), superimposed during voluntary contractions, elicits a motor-evoked potential (MEP) in the surface EMG signal of the target contracting muscle. The MEP amplitude is thought to reflect corticospinal excitability (Rossini et al. 2015), whilst a period of electrical inactivity immediately after the MEP, referred to as the silent period (Damron et al. 2008), is thought to reflect corticospinal inhibitory mechanisms (Säisänen et al. 2008). Despite extensive use of TMS to explore corticospinal excitability/inhibition at the plateau of a maximal voluntary contraction (MVC; Todd et al. 2016), TMS has not been commonly used during the rising torque of explosive voluntary contractions, where RTD is typically measured.

Before using TMS to assess corticospinal excitability/inhibition during explosive contractions, it is important to establish the test–retest reliability of TMS responses (MEP amplitude and silent period) during such conditions; however, this has not been done. In contrast, the test–retest reliability for TMS responses at the plateau of MVCs has been investigated in various muscles. For absolute and normalised MEP amplitude at the MVC plateau in the lower limbs, studies have reported moderate inter-class correlation (ICC; 0.52–0.79) (Mileva et al. 2012; Souron et al. 2016) and coefficient of variation (CV) of 10–11% (Souron et al. 2016). For the silent period at the MVC plateau, the test–retest ICC has ranged from moderate in the vastus lateralis [0.61–0.70; (Di Virgilio et al. 2022)] to excellent in the soleus and tibialis anterior (0.93–0.95; Mileva et al. 2012; Souron et al. 2016) and one study has reported a CV of 8.6% (Souron et al. 2016) in the tibialis anterior. It is unclear whether the reliability of MEP amplitudes and silent periods during explosive contractions will be comparable to that observed at the MVC plateau. Typically, the reliability of torque and EMG amplitude measurements is lower in early compared to later phases of explosive contraction, and generally lower for explosive contractions compared to the MVC plateau (De Ruiter et al. 2004; Buckthorpe et al. 2012), so a similar pattern may be observed for the reliability of MEP amplitudes and silent periods.

TMS responses are typically averaged across multiple separate stimuli (in separate contractions) to minimise the influence of random variation and improve reliability. Thus, when assessing the test–retest reliability of MEP amplitudes and silent periods, it is important to consider how many stimulations might be required to achieve adequately reliable averages. A relatively high number of stimuli (≥ 20) are needed to obtain moderate to good test–retest reliability (ICC = 0.50—0.75;Goldsworthy et al. 2016; Biabani et al. 2018) of MEP amplitudes recorded when the participant is passive. In contrast, as few as four stimuli have been shown to produce good (≥ 0.75) test–retest ICC for MEP amplitude during submaximal contractions held at a constant force (Wheaton et al. 2009; Lewis et al. 2013; Temesi et al. 2017). The effect of the number of contractions on test–retest reliability of average TMS responses has not been determined during either MVC or explosive contractions. Previous studies of TMS responses during MVCs have typically only collected and averaged 3–5 responses (Luc et al. 2014; Tallent et al. 2017; Škarabot Ansdell et al. 2019). This could be due to time constraints or concerns over fatigue with multiple contractions; however, it may be at the expense of poor reliability. Thus, there is a need to assess the effect of the number of contractions on the test–retest reliability of average TMS responses during MVCs and explosive contractions.

In studies of TMS responses, measures are typically averaged across all stimulations obtained within a testing session. However, a subset of contractions are typically used for studies of explosive contractions, with the best three out of ten contractions being recommended for averaging RTD measurements (Maffiuletti et al. 2016). Therefore, for studies of MEPs recorded during explosive contractions, it may be desirable to investigate MEPs obtained from the best three contractions rather than from all contractions, and investigation into the reliability of this approach is necessary.

This study aims to assess the absolute (ICC) and relative (CV) test–retest reliability of TMS responses (MEP amplitude and silent period) recorded at different time points (early, middle, and late phases) during explosive voluntary contractions and at the plateau of MVCs. In so doing, we will also(i) document how the number of consecutive contractions (between 3 and 15) over which TMS responses are averaged, influences test–retest reliability; and (ii) document test–retest reliability of TMS responses averaged across the best 3, out of 10, contractions. We determine the best three as those with the highest torque (Torque averaging method) or EMG RMS (EMG averaging method) prior to MEP on-set.

## Methods

### Participants

Fourteen participants, nine males (age 31 ± 5 years, height, 178.7 ± 6.6 cm, and mass 79.2 ± 4.7 kg) and five females (age 29 ± 6 years, height, 165.5 ± 5.5 cm, and mass 59.3 ± 6.2 kg) were recruited to take part in this study. All participants self-reported to be habitually performing 120–180 min of moderate to high-intensity activity per week, with moderate and high-intensity activity defined according to WHO guidelines (WHO 2016). Participants were also free from injury and disease (screened via a questionnaire adapted from Balady et al. 1998) and free from contraindications to TMS (screened via a questionnaire adapted from Rossi et al. 2011). Due to changes in endogenous hormones throughout the menstrual cycle potentially affecting neuromuscular responses to TMS (Ansdell et al. 2019), female participants undertook experimental trials exclusively during their self-reported early to mid-follicular phase (first ten days from the first day of menstruation), when endogenous hormone concentration is low and relatively stable (de Jonge et al. 2019). The University of Roehampton ethics committee approved the study and all participants provided written informed consent before participating.

### Overview

Participants visited the laboratory on three separate occasions and were asked to avoid strenuous exercise and alcohol consumption for 24 h before each visit. Each session lasted approximately 120–150 min, with consecutive sessions separated by 3–7 days. The first visit was a familiarisation session, and the second and third were measurement sessions. The measurement sessions were performed at a consistent time of day and involved an identical protocol, with measurements used to assess the test–retest reliability of the variables of interest. Each session involved knee extensor torque and EMG measurement during maximal voluntary and explosive contractions, using TMS to obtain superimposed MEP and femoral nerve stimulation to obtain compound muscle action potentials at rest.

### Torque measurements and surface electromyography (EMG)

Participants were tightly secured in a custom-built strength testing chair (Fig. in Maffiuletti et al. 2016) with a waist belt and shoulder straps. The hip and knee angles were set at 100° and 105°, respectively (full extension being 180°). All contractions were isometric knee extensions performed with the right leg. An ankle strap joined to a calibrated S-shaped load cell (FSB-1.5kN, Force Logic, Reading, UK) was secured 4 cm proximal to the medial malleolus. The force signal was amplified (× 375) and then sampled at 2000 Hz (Micro3 1401 and Spike2 v.8; CED., Cambridge, UK). Offline, the force was filtered (fourth-order low-pass Butterworth, 250 Hz cut-off), corrected for limb weight, and multiplied by the external moment arm to calculate joint torque.

The skin was prepared by shaving, cleaning (70% ethanol), and lightly abrading the area where EMG electrodes were placed. A single, bipolar silver-silver-chloride gel-electrode configuration (2-cm diameter and 2-cm inter-electrode distance; Dual Electrode, Noraxon, Arizona, USA) was placed over the belly of each of the rectus femoris (RF), vastus lateralis (VL) and vastus medialis (VM), based on SENIAM guidelines (Hermens et al. 1999). The three wireless EMG signals were filtered (10 Hz, high pass), amplified (× 200) at the source and transmitted to a desktop receiver for further amplification (total system gain × 500; TeleMYO D.T.S., Noraxon, Arizona, USA) and sampled, along with the single wired EMG signal, at 2000 Hz via the same A/D convertor and software as the force signal. However, the EMG system has an inherent 312-ms delay, so whilst suitable for measurements involved in the study, EMG signals sampled by it could not be used to detect activation on-set and trigger the TMS in real-time during the explosive contractions (explained below in Experimental procedures). Thus, a second wired bipolar EMG electrode (2 cm diameter and 2 cm inter-electrode distance; dual Electrode, Biometrics Ltd, Gwent, UK) was placed on the belly of the VM to trigger the TMS during contraction. Electrode locations were marked with a permanent marker pen, and participants were asked to maintain these marks throughout the study by re-applying them if necessary. Once offline, wireless EMG signals were filtered (fourth-order Butterworth, band-pass, 6–500 Hz) and time-corrected for the 312-ms delay inherent in the Noraxon system.

### Transcranial magnetic stimulation (TMS)

TMS with a 1-ms pulse width was delivered via a double cone coil (110-mm Magstim 200, Whitland, UK) over the scalp in an optimal position to elicit MEPs in the right quadriceps muscles. The following procedures were completed in every session. Participants wore a swim cap, and the vertex of the head—identified as 50% of the distance between (i) nasion and inion and (ii) right and left perpendicular point—was marked on the swim cap. A 5-by-5-cm grid with 1-cm spacing between grid lines was drawn on the swim cap, lateral (left hemisphere) and posterior from the vertex. The coil was moved posteriorly and laterally from the vertex in ~ 0.5-cm steps, and in each position, the participant completed four submaximal voluntary contractions at 20% MVC torque (established in the warm-up; see below) with superimposed TMS on each contraction at a submaximal (range 50–60%) stimulator output. The position which produced the highest consistent MEP amplitudes (peak-to-peak) over the four superimposed contractions for all three muscles (RF, VL and VM) was deemed the optimal coil position. Once established this was marked by drawing the edge of the coil over the swim cap and used throughout the remainder of the session. The active motor threshold (AMT) was then determined via a series of 20% MVC torque contractions superimposed with TMS stimulator output starting at 39%. The AMT was defined as the minimum TMS intensity required to elicit five visible MEPs amongst the background EMG activity of the VM and RF, out of ten consecutive superimposed contractions. If the muscles had less or more than five visible MEPs, the machine intensity was reduced or increased by 2% of the machine output. We prioritised the VM and RF (not the VL) as these muscles produced more consistent and visible MEPs in pilot testing. Where it was impossible to match AMT for VM and RF, we settled on being one visible MEP away from 5/10 (e.g., 6/10 VM and 4/10 RF). The same investigator held the coil by hand throughout the measurement sessions, continuously monitoring its position and orientation. For TMS delivered during maximal and explosive contractions (see experimental protocol), the intensity was set at 140% of the stimulator output at AMT (Groppa et al. 2012; Rossini et al. 2015; Rossi et al. 2020).

### Femoral nerve electrical stimulation

Single, square-wave pulses (200 µs duration) were delivered (DS7AH, Digitimer, Hertfordshire, UK) over the femoral nerve in the inguinal triangle to evoke twitch contractions and obtain compound muscle action potentials (M-waves) at rest. The anode (5 × 8 cm carbon rubber; EMS Physio Ltd., Oxfordshire, UK) was placed over the head of the greater trochanter. The optimal location of the cathode (1 cm diameter tip; S1 Compex Motor PointPen, Digitimer, UK) was determined as that which evoked the greatest peak twitch torque for a given submaximal stimulation intensity (80–120 mA). The cathode was taped down and held in position by the same investigator as a series of twitches at incremental intensities were evoked until there was a plateau in the peak-to-peak M-wave amplitude (M_max_) of all three muscles (RF, VL, VM). The stimulator intensity was then increased to 150% of the intensity at M_max_, ensuring supramaximal intensity. Three supramaximal twitch contractions were then evoked, each separated by 15 s, and M_max_ was averaged across the three contractions for each muscle. The procedures were repeated for all sessions.

### Experimental protocol

**Fig. 1:**
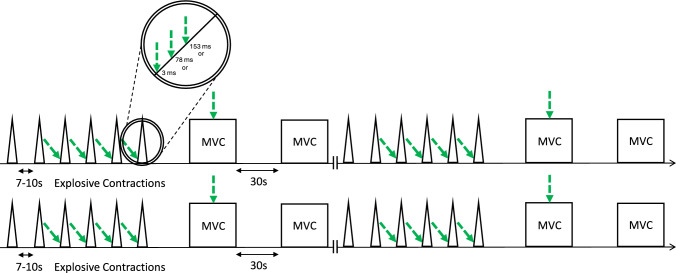
Schematic depicting a single experimental block, of which there were three for each experimental session. Each triangle represents an explosive contraction, while each square represents a MVCs. Each green dashed arrow represents a superimposed TMS. Each block differed in timing of the TMS during the explosive superimposed contractions, as shown in the insert. For a clearer visual of the timing of the TMS during the different explosive contractions, relative to EMG onset, please see Fig. 2

**Fig. 2:**
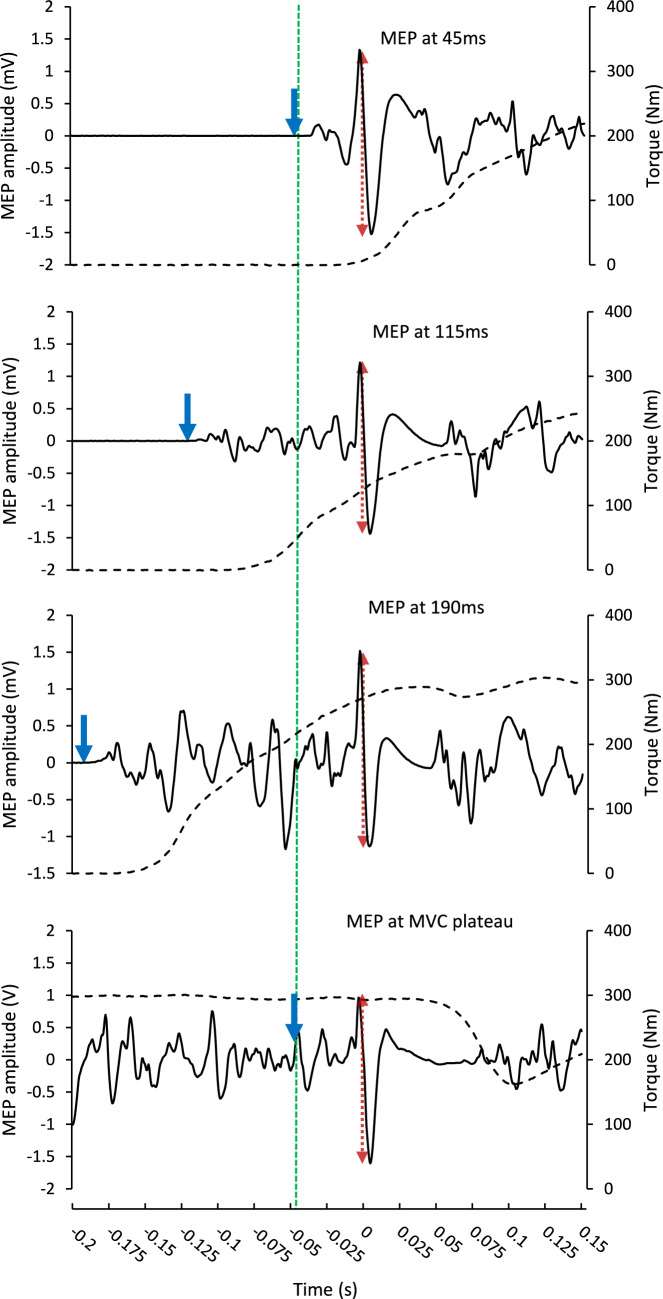
Representative traces of motor evoked potentials (full black line) in the vastus medialis and knee extensor torque (dashed line) in each of the four conditions. The downward pointing blue arrows indicate EMG on-set for the three explosive contraction phases (top three plots) and manual TMS trigger time for the MVC phase (bottom plot). The long green dashed line represents the TMS stimulation in each contraction phase. The labels in the top three plots reflect approximate MEP times relative to EMG on-set. The red dotted arrows represent the peak-to-peak amplitude of the MEPs used for analysis. Zero seconds on the x-axis represents the midpoint of the MEP where it crosses from a positive to negative value (or vice versa)

Participants first completed a warm-up involving a series of explosive, submaximal, and maximal voluntary contractions (the latter being used to establish MVC torque), followed by procedures for obtaining optimal TMS coil position, ATM, and M_max_. Participants then completed a series of MVCs and explosive voluntary contractions with and without superimposed TMS. The instruction for MVCs was to “push as hard as possible” for 3–5 s and explosive contractions to “push as fast and hard as possible”, emphasising fast for 1 s. The protocol was organised into three blocks of contractions. Each block (Fig. 1) involved 24 explosive contractions (8 without and 16 with superimposed TMS) and 8 MVCs (3 without and 5 with superimposed TMS), distributed across four sets. Each set involved six explosive contractions (the final four with TMS stimulation) and two MVCs. In sets 1–3, one of the two MVCs had superimposed TMS (randomly ordered), and in set four, both MVCs had superimposed TMS. Participants rested 10 s between explosive contractions, 30 s between MVCs, 120 s between sets, and 300 s between blocks. Each block was identical except for the timing of TMS application during explosive contractions, with each block using a different TMS timing condition for these contractions (see next section). The order of the blocks was randomised across participants but held constant across sessions for the same participant. Overall, the 3-block protocol yielded 24 explosive contractions and 9 MVCs without TMS, and 48 explosive contractions (16 per stimulus time condition) and 15 MVCs with TMS Fig. 2.

For superimposed MVCs, the TMS was triggered manually by the same experienced investigator during the torque plateau of the MVC. For superimposed explosive contractions, the TMS was triggered automatically when the VM EMG signal of the wired system exceeded, above or below, a set threshold. The threshold was set in the Spike2 software as the lowest amplitude for that session above the highest peaks and troughs of the baseline noise. TMS was triggered at 3, 73, or 148 ms from EMG threshold crossing for early, middle, and late-phase conditions, respectively. When expressed relative to manually detected EMG on-set (determined via the methods of Tillin et al. 2010) TMS triggers occurred at approximately 8 (early), 78 (middle), and 153 (late) ms owing to manually detected on-set preceding threshold crossing by approximately 5 ms. The centre of the resulting MEPs occurred at approximately 45 (early), 115 (middle), or 190 ms (late) from manually detected EMG on-set (Fig. 2).

### Data screening and analysis

Out of 15 superimposed MVCs, only those where the TMS was delivered > 90% of MVC torque were used for further analysis. Out of the 16 superimposed explosive contractions per condition, only those that met the following criteria were used for further analysis: (i) average baseline force did not change by > 2 Nm during the 200 ms preceding manually detected force on-set (detected as in Tillin et al. 2010); and (ii) there was a genuine attempt at an explosive contraction. A genuine attempt at an explosive contraction was defined as the instantaneous slope of torque-time, just prior to MEP on-set, being within three standard deviations (SD) of mean instantaneous slope at the same time point for contractions without TMS. The time points for measuring instantaneous slope were 30, 105, and 180 ms after manually detected torque on-set, for early-, middle- and late-phase conditions, respectively. Given the criteria above, the maximum number of MEPs analysed further in each condition was limited to the number of usable contractions in the participant with the lowest number of usable contractions. This was 9 (early), 10 (middle), and 15 (late) for the explosive contractions, and 15 for the MVCs.

**Fig. 3:**
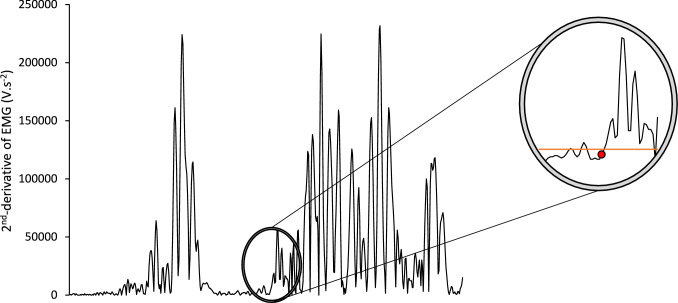
Example of how the silent period offset was detected in the VM of one participant. The data shows the second derivative of the EMG-time trace after rectification. Circled data are an example of SP offset detection. The orange line represents the detection threshold (5 SD of the mean baseline), whilst the red dot, which is the detected offset, represents the last time point before the EMG signal crosses threshold, and remains above the threshold for ≥ 70% of the data points recorded in the next 10 ms

For useable contractions, MEP amplitude was defined as the peak-to-trough of the superimposed response (Fig. 2) and is reported in absolute terms and normalised to M_max_. The silent period was measured from the point of stimulation to the resumption of EMG activity after the MEP offset. To determine the resumption of EMG activity, the second derivative of EMG amplitude over time was established (1-ms time constant) and then the signal was rectified. EMG activity resumption was defined when the amplitude of the second derivative increased above 5 SD of the mean baseline (calculated in a 500-ms period prior to the contraction) for 70% of the next 10 ms (adapted from Damron et al. 2008; Fig. 3). This automated process was confirmed via manual inspection of the signal as recommended by Damron et al., (2008). MEP amplitude (absolute and normalised to M_max_ separately) and silent periods were extracted on a muscle level before being averaged across the three muscles to obtain a quadriceps mean. This is standard practice in studies looking to relate general quadriceps EMG data to net knee extensor torque and RTD (Tillin et al. 2010, 2012, 2013, 2018; Folland et al. 2014; Behrens et al. 2015; Morales-Artacho et al. 2018; Cossich and Maffiuletti 2020).

### Averaging methods

For each condition (early, middle, and late explosive phases, and at MVC plateau), mean quadriceps consecutive running averages of the dependent variables were calculated from the first three stimuli/contractions, up to the maximum number of usable contractions: 9 (early), 10 (middle), and 15 (late) for the explosive contractions, and 15 for the MVCs. Dependent variables also were averaged over the best 3 out of the first 10 contractions in each condition (9 for early-phase explosive), using two different methods to define best contractions. For the torque averaging method, the best three explosive contractions were those with the greatest torque just prior to MEP (i.e., at 30, 105, and 180 ms from torque on-set, in early, middle, and late conditions, respectively), whilst the best 3 MVCs were those with the highest average torque over 1 s prior to the MEP. For the EMG averaging method, the best explosive contractions were those with the highest EMG RMS amplitude (averaged of all three muscles) from on-set to the start of the MEP (i.e., 0–23, 0–98, 0–173 ms, in early, middle, and late, respectively), whilst the best three MVCs were those with the greatest EMG RMS in the 1 s period before MEP.

### Statistical analysis

To check for any fatigue within or between sessions, paired t-tests were used to compare the highest MVC torques between the first set of the first block and the last set of the last block, within each session separately, and to compare the highest MVC torques between sessions.

Paired t-tests were used to compare the differences between the test and re-test sessions within each condition for each dependent variable. The relative reliability of TMS responses was assessed via the relative test–retest interclass correlation coefficient (ICC) determined via a two-way mixed-effects model, with the absolute agreement, as suggested for a test–retest design (Koo and Li 2016). ICCs were interpreted as < 0.5 poor, ≥ 0.5 but < 0.75 moderate, ≥ 0.75 and < 0.9 good, and ≥ 0.9 excellent (Koo and Li 2016). The coefficient of variation (CV) determined the absolute reliability (degree of fluctuation of repeated measurements within individuals) of dependent variables and was calculated for each participant (100 * test–retest standard deviation (SD)/test–retest mean) before being averaged across participants.

Separate ICCs and CVs were produced for each averaging approach; consecutive running averages and the best three based on both torque and EMG averaging methods. Logarithmic functions ($y=a\text{ln}(x)+\text{k}$) were fit to data describing the relationship between ICC or CV and the number of consecutive contractions/stimuli over which the dependent variables were averaged to establish trends in the data and enable extrapolation of the ICC or CV beyond the maximum number of useable contractions. Only statistically significant (*p* < 0.05) logarithmic functions are reported in the results. Logarithmic functions were chosen as they provided excellent fits with significant (*p* < 0.05) relationships (*r*^2^ = 0.97–0.75 and SD = 0.08) and did not cross zero values of CV (as with, e.g., exponential fits), which are physiologically impossible. Best-fit logarithmic functions were made in SPSS v.26 (IBM Inc., Armonk, NY, USA). For ICCs, a confidence interval of 95% was used for the lower bound (Lb) and upper bound (Ub), and CVs were calculated using MATLAB (R2021a Update 5, MathWorks Inc., Natick, MA, USA). The significance level was set at *p* ≤ 0.05. Data are presented in the results as mean ± SD.

## Results

There was no difference in MVC torque between session 1 and 2 (− 0.8 ± 5.1%; *p* = 0.376) suggesting that there were no fatiguing effects following session 1. Within sessions, MVC torque declined from the start to the end of session 2 (− 5.5 ± 8.5%; *p* = 0.048) but not session 1 (− 4.3 ± 11%; *p* = 0.221), suggesting there was minimal within session fatigue overall.

There was no significant difference (*p* ≥ 0.168) between the test and retest sessions for any of the dependent variables in each condition Table(1).

**Table 1:** Absolute and normalised MEP amplitude, and silent period for each condition in each session (test and re-test)

	MEP amplitude (mV)		MEP amplitude (% M_max_)		Silent Period (s)	
	Test	Re-test	Test	Re-test	Test	Re-test
Early	0.96 ± 0.40	0.92 ± 0.38	30.2 ± 8.3	30.2 ± 9.4	0.078 ± 0.017	0.078 ± 0.013
Middle	0.97 ± 0.47	0.95 ± 0.40	30.0 ± 6.9	31.7 ± 7.8	0.083 ± 0.019	0.083 ± 0.015
Late	1.01 ± 0.45	0.96 ± 0.41	31.8 ± 7.7	31.3 ± 6.9	0.088 ± 0.016	0.089 ± 0.014
MVC	1.10 ± 0.46	1.02 ± 0.39	35.4 ± 7.3	35.5 ± 10.3	0.091 ± 0.011	0.093 ± 0.010

Normalised MEP amplitude is a % of maximal M-wave (M_max_). Conditions are early (45), middle (115), and late (190) phases of explosive contractions, and at the MVC plateau. Data are group mean ± SD of the first 10 (9 for early) contractions performed in each condition

### Reliability as a function of the number of MEPs

**Fig. 4:**
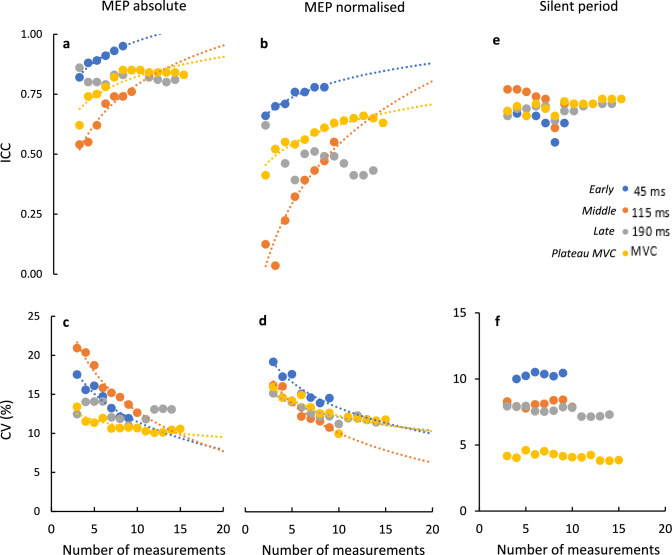
Test–retest ICC (**A**, **B**, **E**) and CV (**C**, **D**, **F**) for dependent variables as a function of the number of consecutive contractions over which data are averaged. Dependent variables are absolute (A and C) and normalised (B and D) MEP amplitude, and silent period (E and F). For normalised data, MEP amplitude is normalised to maximal M-wave. MEP amplitudes and silent periods were recorded at the plateau of MVCs, and at three different time points from activation on-set during explosive contractions: early (45 ms), middle (115 ms), and Late (190 ms). Logarithmic functions (y = a ln(x) + k) were fitted to data and are plotted as a dotted line for conditions where the fit was significant (*p* < 0.05)

The test–retest ICCs for absolute and normalised MEP amplitude generally increased with the number of averaged MEPs (Fig. 4A, b), whilst the test–retest CV for the same variables generally decreased (Fig. 4C, D). These trends indicate increasing test–retest MEP amplitude reliability with increasing numbers of averaged MEPs. The exception to these general trends was the late phase. In this condition, although the CV of normalised MEP amplitude was related to the number of averaged MEPs (Fig. 3D), the CV of absolute MEP amplitude, the ICC of absolute MEP amplitude, and the ICC of normalised MEP amplitude were not related to the number of averaged MEPs (Fig. 4A, B, C). These trends, apparent upon visual inspection, were supported by logarithmic fits to the data, which were significant in all cases aside from the late phase cases highlighted in the previous sentence (Table 2).

**Table 2:** Coefficients (a and k), the goodness of fit (R^2^) of the logarithmic functions $y=a\mathit{ln}(x)+k$

ICC											
MEP absolute	a	k	*R*^2^	*p*	n of	MEP normalised	a	k	*R*^2^	*p*	n of
Early	0.116	0.704	0.96	0.001	2	Early	0.115	0.537	0.95	0.001	7
Middle	0.231	0.265	0.92	0.001	9	Middle	0.392	−0.395	0.84	0.002	19*
Late	0.002	0.819	− 0.20	0.950	–	Late	− 0.093	0.660	0.13	0.220	−
MVC	0.427	0.200	0.93	0.001	4	MVC	0.154	0.273	0.85	0.002	23*

CV											
MEP absolute	a	k	*R*^2^	*p*	n of	MEP normalised	A	k	*R*^2^	*p*	n of
Early	− 5.17	23.38	0.90	0.001	14*	Early	− 4.77	24.28	0.87	0.001	20*
Middle	− 7.23	29.55	0.94	0.001	15*	Middle	− 5.41	22.61	0.93	0.001	11*
Late	− 1.76	15.82	0.16	0.210	–	Late	− 3.04	18.71	0.95	0.001	18*
MVC	− 2.16	15.19	0.68	0.013	11	MVC	− 2.90	19.04	0.79	0.005	23*

Functions were fit to describe the relationship between test–retest ICC or CV and the number of consecutive contractions over which MEP amplitudes were averaged. The ‘n of’ is the number of MEP amplitudes averaged to reach ICC ≥ 0.75 or CV ≤ 10%. The * denotes where ‘n of’ has been extrapolated beyond the measured data. MEP amplitudes were recorded at the plateau of MVCs, and at three different time points from EMG on-set during explosive contractions: early (45 ms), middle (115 ms), and late (190 ms)

ICCs were generally higher for absolute MEP amplitude (Fig. 4A) than normalised MEP amplitude (Fig. 4B). For absolute MEP amplitude, good ICC (ICC > 0.75) was found within just three averaged MEPs for all conditions except middle (where it was attained within nine averaged MEPs). For normalised MEP amplitude, good ICC was only achieved in the early condition (attained within seven contractions), whilst other conditions only reached moderate ICCs (ICC > 0.50). CV tended to be similar for absolute and normalised MEP amplitudes, reaching 10–15% values for all conditions within seven contractions (Fig. 4C, D). We were interested in identifying the number of averaged MEPs needed to attain good ICC (ICC > 0.75) and CV < 10% for all variables and conditions. As is noticeable from Fig. 4A–D, this was not consistently reached using the number of MEPs measured in this study (9–15, depending on the condition). Therefore, the fitted logarithmic functions provided estimates by extrapolating beyond fitted data where necessary (see Table 2 “n of” columns).

In contrast to the general trends for MEP amplitude reliability, the silent period reliability did not vary as a function of number of averaged MEPs (Fig. 4E,F). In support, attempted logarithmic fits were all non-significant (*p* > 0.05; fits not shown). Moderate ICCs (0.5 ≤ ICC ≤ 0.75) were generally observed for the silent period in all conditions (Fig. 4E). CVs for the silent period (Fig. 4F) were lower than for MEP amplitude, particularly during MVC.

### Reliability for best three contractions using EMG or torque methods

Compared to averaging the first 10 consecutive contractions, the ICCs overall tended to be lower (− 0.07 ± 0.01) and CVs overall slightly higher (+ 1.7 ± 0.2%) for either of the best 3 averaging methods, across all conditions and dependent variables (Table 3). There was no consistency for the torque (best 3) averaging method to be better than the EMG averaging method, nor vice versa, with the higher ICC and lower CV between methods being dependent on condition and variable Table 4.

**Table 3:** Test–retest ICC, with 95% confidence interval in parenthesis, of dependent variables determined via three different averaging methods: average of the first ten consecutive contractions, and average of the best three based on those contractions with the highest EMG or torque prior to the MEP

Dependent variables	Averaging method		
Absolute MEP amplitude	First 10	Best 3	
		EMG	Torque
Early	0.95 (0.84–0.98)	0.93 (0.79–0.98)	0.88 (0.65–0.96)
Middle	0.79 (0.47–0.93)	0.74 (0.26–0.89)	0.69 (0.64–0.97)
Late	0.85 (0.61–0.95)	0.83 (0.56–0.94)	0.84 (0.57–0.94)
MVC	0.85 (0.59–0.95)	0.60 (0.14–0.85)	0.77 (0.43–0.92)
Normalised MEP amplitude	First 10	Best 3	
		EMG	Torque
Early	0.78 (0.39–0.92)	0.72 (0.34–0.90)	0.65 (0.21–0.87)
Middle	0.55 (0.06–0.83)	0.37 (−0.1–0.74)	0.28 (−0.3–0.69)
Late	0.49 (−0.05–0.81)	0.49 (−0.07–0.80)	0.40 (−0.17–0.76)
MVC	0.63 (0.14–0.86)	0.53 (0.02–0.82)	0.72 (0.32–0.90)
Silent period	First 10	Best 3	
		EMG	Torque
Early	0.62 (0.14–0.88)	0.49 (0.15–0.88)	0.64 (−0.2–0.92)
Middle	0.71 (0.28–0.90)	0.63 (0.21–0.89)	0.68 (0.16–0.93)
Late	0.68 (0.21–0.89)	0.63 (0.13–0.87)	0.63 (0.13–0.87)
MVC	0.71 (0.23–0.89)	0.66 (0.20–0.88)	0.77 (0.42–0.92)

Dependent variables are absolute MEPs, normalised MEPs (normalised to maximal M-wave), and silent period. MEP data were recorded at the plateau of MVlCs, and at three different time points from EMG on-set during explosive contractions: early (45 ms), middle (115 ms), and late (190 ms)

**Table 4:** Test–retest CV mean ± SD of dependent variables determined via three different averaging methods: average of the first ten consecutive contractions, and average of the best three based on those contractions with the highest EMG or torque prior to the MEP

Dependent Variables	Averaging method		
Absolute MEP amplitude	First 10	Best 3	
		EMG	Torque
Early	11.7 ± 10	11.9 ± 9.6	16.5 ± 14.6
Middle	12.6 ± 8.9	15.5 ± 11	14.3 ± 10.1
Late	10.7 ± 7.8	12.6 ± 8.5	12.6 ± 8.2
MVC	10.7 ± 6	14.7 ± 11.9	10.0 ± 10.1
Normalised MEP amplitude	First 10	Best 3	
		EMG	Torque
Early	14.0 ± 9.8	11.9 ± 10.6	16.9 ± 14.3
Middle	10.0 ± 7.8	12.3 ± 9.9	13.4 ± 11
Late	11.0 ± 5.1	12.1 ± 7.6	13.1 ± 7.8
MVC	10.0 ± 7.2	17.1 ± 10.3	13.1 ± 9.3
Silent period	First 10	Best 3	
		EMG	Torque
Early	9.7 ± 9.2	10.3 ± 9.3	8.2 ± 7.3
Middle	7.9 ± 7.1	9.4 ± 7.3	9.0 ± 7.2
Late	7.8 ± 5.7	8.5 ± 5.2	8.9 ± 5.3
MVC	4.1 ± 3.4	4.7 ± 4.1	4.3 ± 2.4

Dependent variables are absolute MEPs, normalised MEPs (normalised to maximal M-wave), and silent period. MEP data were recorded at the plateau of MVCs, and at 3 different time points from EMG on-set during explosive contractions: early (45 ms), middle (115 ms), and late (190 ms)

### Reliability at single muscle level

It was beyond the scope of this study to assess test–retest reliability of TMS responses in the separate muscles. However, for the readers interest we have provided the ICC and CV data for each muscle, next to the average of the quadriceps, in supplementary data Tables 2 and 3.

## Discussion

We investigated the absolute (ICC) and relative (CV) test–retest reliability of TMS responses (MEP amplitude and silent period duration) recorded at different time points (early, middle, and late) during explosive voluntary contractions, and at the plateau of MVCs. In general, no one condition (explosive early, middle, late, or MVC plateau) produced consistently more reliable (absolute or relative) TMS responses across the different averaging methods than another. In all conditions except the explosive late phase, both absolute and relative reliability of MEP amplitude (absolute and normalised) generally increased with an increase in the number of consecutive stimuli (separate contractions) averaged. In contrast, increasing the number of averaged stimuli did not improve silent period reliability in any condition. This study also found averaging the best 3 out of the first 10 contractions based on EMG amplitude or torque output, produced comparable, albeit slightly poorer, reliability than averaging the same 10 contractions for all TMS responses and conditions.

### Reliability during MVCs

To date, only a handful of studies have investigated the test–retest reliability of MEPs evoked at the MVC plateau, and these have all been based on measures averaged across 3–5 MVCs (Kamen 2004; Mileva et al. 2012; Souron et al. 2016). For comparison within this section, we will focus on our findings within this range of 3–5 contractions, with the influence of higher numbers of contractions being discussed later.

Within 3–5 MVCs, we observed moderate ICC (0.50–0.75) for absolute and normalised MEP amplitudes, which is within the range (0.47–0.79) of what others have observed (Kamen 2004; Sidhu et al. 2009; Mileva et al. 2012; Souron et al. 2016; Table 1 in supplementary material). We also observed moderate ICC for silent periods at MVC plateau (0.66–0.70) which was comparable to what has previously been reported for the rectus femoris (0.61–70; Di Virgilio et al. 2022) and lower than reported for the tibialis anterior (0.93–0.95; Mileva et al. 2012; Souron et al. 2016; Table 1 in supplementary material). For test–retest CV, within 3–5 MVCs, we observed 11–16% values for absolute and normalised MEP amplitude and below 5% for the silent period. One previous study has reported test–retest CV for TMS responses at MVC plateau in the tibialis anterior (Souron et al. 2016) and reported slightly lower CVs for MEP amplitude (10–11%) and a slightly higher CV for silent period (9%), compared to our results for the quadriceps. A more detailed comparison of our results and those of previous studies is difficult because of the disparity in methods for factors such as the type of ICC used, muscles involved, TMS intensity used, and method of calculating silent period. Nevertheless, based on our results and those of previous studies, averaging data across only 3–5 MVCs should provide moderate ICCs and CVs < 16% for MEP amplitudes and silent periods.

Independently of the number of averaged MEPs, absolute MEP amplitude (V) at MVC plateau showed better ICC values than normalised MEP amplitude (% M_max_), and this pattern was consistent for other conditions also. In contrast, a previous study of the tibialis anterior (Souron et al. 2016) found ICC for MEP amplitude at the MVC plateau to be similar between absolute and normalised data. This difference between our study and Souron et al. (2016) may be due to the different muscle groups used, although there were other methodological differences (e.g., process for determining coil position and intensity), again making a direct comparison difficult. The greater ICC in absolute compared to normalised MEP amplitude that we observed might originate from the ICC calculation itself. ICC scores will be influenced by inter-participant variability, with greater variability likely contributing to greater ICCs (Koo and Li 2016). Normalising the MEP amplitudes to M_max_ will control for some of the factors that cause inter-participant variability, such as muscle size and adiposity (Besomi et al. 2020), which in turn might reduce ICCs. Evidence for this is presented in Table 1, which shows group SDs relative to group means to be ~ 40% lower for normalised vs absolute MEP amplitudes. Another factor may be the reliability of the M_max_ which, consistent with the literature (Place et al. 2007) was only moderate (ICC = 0.67), so may have contributed to reducing the ICC of the MEP after normalising to M_max_. In contrast to ICC, CV measurements are not influenced by inter-participant variability which may explain why CVs were generally similar for absolute and normalised MEP amplitudes.

### Reliability during explosive contractions

We had suspected the reliability of TMS responses to be lowest in early-phase explosive contractions and highest at the MVC plateau, as this is commonly observed for torque and EMG amplitudes in these different conditions (Folland et al. 2014; Tillin and Folland 2014). However, our findings indicated the test–retest reliability of TMS responses was similar for the different conditions, an observation that appeared unrelated to the number of contractions averaged.

The only exception to there being no clear differences in reliability between conditions was the observation that CV for the silent period was generally highest for the early phase and lowest for the MVC plateau (Fig. 2F). This may be associated with the lower overall length of the silent period in the early phase compared to the MVC plateau (Table 1). Given that the CV is a measure of variability relative to the mean, a condition with a lower mean silent period (as is the case for the explosive early phase) will be susceptible to higher CV, assuming a comparable between-session SD. The shorter silent period in the early phase may be due to neural drive in this phase being predominantly feedforward (Škarabot et al. 2021) and thus less affected by afferent feedback. Further, the short (78 ms) silent period is likely due to inhibition being caused at a spinal level (Škarabot Mesquita et al*.* 2019), as opposed to intracortical inhibition which lengthens the silent period (Fuhr et al. 1991).

### Number of averaged MEPs

For absolute and normalised MEPs, ICCs generally increased, and CVs decreased, with an increasing number of contractions, in all conditions except the late phase. This is in agreement with studies on MEP amplitudes recorded at rest or at submaximal force, which also observed improved reliability by increasing the number of averaged stimuli (Goldsworthy et al. 2016; Biabani et al. 2018; Brownstein et al. 2018). Our results show that the conditions in our study, except for the late-explosive phase, require 11–15 contractions (absolute MEP amplitude) and 18–23 contractions (normalised MEP amplitude) to ensure both good ICCs (≥ 0.75) and CVs ≤ 10%, or 7 contractions (absolute and normalised MEP amplitude) for moderate ICCs (≥ 0.5) and CVs ≤ 15%. Further, although caution is required when extrapolating the logarithmic functions reported in this study well beyond the number of contractions measured, the shape of the logarithmic functions indicates little benefit to reliability will be gained by sampling beyond the abovementioned ranges. Variability in the MEP responses in our study are likely affected by the TMS intensity we used (140% AMT at 20% MVC), which evokes MEPs in the quadriceps half-way up the impulse-response relationship for the quadriceps (Temesi et al. 2017). As this is the steepest section of the impulse-response relationship, small changes in corticospinal excitability can greatly affect MEP amplitude (Temesi et al. 2017). However, it is important to note the steepest section of the impulse-response relationship at 20% MVC may not reflect the same section of the impulse-response curve of MVCs and explosive contractions, which we cannot verify with our current methodology. These issues, combined with the intrinsic fluctuations in ongoing oscillatory activity within the cortical area (Hordacre et al. 2017), likely explain the inherent variability in MEP amplitudes, which seems to limit test–retest reliability, even with consecutive averaging. Nevertheless, based on our results, we recommend future investigators consider collecting and averaging MEP amplitudes across more than the standard 3–5 MVCs (or explosive contractions) used in previous studies, if this can be managed within their protocol, with an optimal range likely falling between 10 and 20.

Unlike the other conditions, MEP amplitude reliability (ICC and CV) in the late-explosive phase (190 ms) was not improved by increasing the number of averaged contractions. We can only speculate about the physiological factors explaining this observation. However, potentially afferent feedback, which will be greater in the later than earlier phases of explosive contraction (Škarabot et al. 2021), inhibits the neural drive to muscle and so might limit the range and thus variability of possible MEP amplitudes in the late phase of explosive contraction.

In the silent period, ICC and CV remained constant despite increases in the number of averaged stimuli. One of the factors contributing to silent period variability is the intensity of stimulation, with lower intensities resulting in higher variability (Damron et al. 2008). Thus, had we used a lower stimulation intensity we may have observed poorer silent period reliability that is improved with an increased number of averaged contractions. The short silent periods we observed (< 100 ms), which imply inhibitions are spinal rather than cortical, may also have contributed to our observations (Škarabot Mesquita et al*.* 2019). Specifically, the spinal inhibitions, unlike cortical inhibitions, may have limited silent period variability to a narrow range of possibilities. Nevertheless, our results show for 140% AMT, averaging data across three explosive contractions or MVCs provides sufficient reliability in silent period that will not improve by increasing the number of averaged contractions.

In studies on explosive contractions, RFD is often averaged from the best 3 out of as many as 10 or more contractions (Maffiuletti et al. 2016). Thus, if intending to relate TMS responses measured during explosive contractions to RFD, it may be pertinent to also average the TMS responses in the best 3 contractions. Of the two methods we used for determining the best 3 (out of the first 10) contractions (torque and EMG methods), neither showed consistently better reliability than the other across all conditions and variables. However, both methods for all conditions provided comparable ICCs and CVs to the average of the first 10 contractions (Tables 3 and 4). It therefore appears that selecting the best 3 contractions to average MEP amplitudes minimises the influence of MEP variability as effectively as increasing the number of averaged stimuli over consecutive contractions from 3 to 10. Thus, averaging the best three contractions may be a viable option for investigators in the future, particularly if looking to relate MEP amplitude measurements to RTD.

## Conclusion

Regardless of the averaging method or reliability metric (ICC and CV), no specific condition produced consistently more reliable MEP amplitudes (absolute or normalised), and silent periods than another. Generally, increasing the number of averaged consecutive contractions increased the reliability for MEPs amplitude (absolute and normalised) in all conditions except for late explosive phase, but did not improve the reliability of silent period duration. Thus, for MEP amplitude, we recommend collecting more than the typical 3–5 contractions, as used by previous studies involving TMS during MVC, and ideally between 10 and 20 contractions, if the study design can accommodate this. However, 3–5 contractions will suffice for silent period measurements. Alternatively, investigators could opt to average the best 3 contractions based on those with the highest torque or EMG prior to MEP, which provides comparable reliability to averaging the first 10 contractions.

## Supplementary Information

Below is the link to the electronic supplementary material.

Supplementary file1 (PDF 188 KB)

Supplementary file2 (PDF 190 KB)

## Data Availability

The data collected and analysed during this study are available from the corresponding author upon reasonable request.

## Abbreviations

AMT: Active motor threshold
CV: Coefficient of variance
EMG: Electromyography
ICC: Inter-class correlation
MEP: Motor-evoked potential
MVC: Maximal voluntary contraction
MU: Motor unit
RF: Rectus femoris
RMS: Root mean squared
RTD: Rate of torque development
SD: Standard deviation
SP: Silent period
TMS: Transcranial magnetic stimulation
VM: Vastus medialis
VL: Vastus lateralis
