# Supplementary material for: Test–retest reliability of TMS motor evoked responses and silent periods during explosive voluntary isometric contractions
Source: Eur J Appl Physiol. 2025 Feb 22;125(7):1841–54. doi: 10.1007/s00421-025-05707-3 (PMC12227505; doi:10.1007/s00421-025-05707-3)
Supplement: Supplementary file 1 — Supplementary file1 (PDF 188 KB) [file 421_2025_5707_MOESM1_ESM.pdf]

Table 1 Between-session ICC for MEP measures evoked and averaged across 3-5 MVCs from current and prior studies.

Study	Muscle/s	Number of MEPs	Between-session ICC			
			ICC Type	Absolute Amplitude	Normalised Amplitude	Silent Period
Current study	Average of VM, VL, RF	3 to 15	3,1 mixed	0.62 [3][a]<br>0.74 [4]<br>0.75 [5]	0.41 [3]<br>0.52 [4]<br>0.55 [5]	0.68 [3]<br>0.70 [4]<br>0.66 [5]
Kamen et al (2004)	BB	5	Not stated	0.68	-	-
Sidhu et al (2009)	RF	4	2,1	-	0.69	-
Mileva et al (2012)	TA[b]	3	1,k	0.79	-	0.93
Souron et al (2016)	TA	3	Not stated	0.47	0.52	0.95
Di Virgilio et al (2022)	RF	3	2,1	-	-	0.7 (general population)<br>0.61 (soccer player)

ICC for Normalised MEP amplitude was also reported in Malcolm et al. 2021, but the reported value (0.59) seems to be averaged across multiple contraction intensities (not limited to MVC).

Key: *[a] Values in square brackets for the current study represent the number of averaged MEPs used to obtain the reported ICCs. [b] study also includes MEPs from Soleus. It is not included here as these were obtained whilst acting as an antagonist.*
Abbreviations: *BB, Biceps brachii; TA, Tibialis anterior, RF, Rectus femoris; VM, Vastus medialis; VL, Vastus lateralis.*
