# Supplementary material for: Test–retest reliability of TMS motor evoked responses and silent periods during explosive voluntary isometric contractions
Source: Eur J Appl Physiol. 2025 Feb 22;125(7):1841–54. doi: 10.1007/s00421-025-05707-3 (PMC12227505; doi:10.1007/s00421-025-05707-3)
Supplement: Supplementary file 2 — Supplementary file2 (PDF 190 KB) [file 421_2025_5707_MOESM2_ESM.pdf]

Table 2 supplementary. Between session ICCs for absolute and normalised MEP and SP measures evoked and averaged across 10 explosive contractions from current study with individual muscle and whole quadriceps.

Absolute MEP amplitude		*VM*	*VL*	*RF*	*Whole Quad*
Early	*ICC*	0.81	0.85	0.86	0.95
Middle	*ICC*	0.64	0.41	0.87	0.79
Late	*ICC*	0.73	0.74	0.70	0.85
Plateau MVC	*ICC*	0.95	0.73	0.61	0.85
Normalised MEP amplitude		*VM*	*VL*	*RF*	*Whole Quad*
Early	*ICC*	0.71	0.62	0.73	0.78
Middle	*ICC*	0.32	0.63	0.54	0.55
Late	*ICC*	0.33	0.37	0.42	0.49
Plateau MVC	*ICC*	0.60	0.53	0.28	0.63
Silent Period		*VM*	*VL*	*RF*	*Whole Quad*
Early	*ICC*	0.62	0.67	0.71	0.62
Middle	*ICC*	0.83	0.82	0.86	0.71
Late	*ICC*	0.77	0.80	0.73	0.68
Plateau MVC	*ICC*	0.73	0.69	0.72	0.71

Table 3 supplementary. Between session CVs for absolute and normalised MEP and SP measures evoked and averaged across 10 explosive contractions from current study with individual muscle and whole quadriceps.

Absolute MEP amplitude		*VM*	*VL*	*RF*	*Whole Quad*
Early	*Mean CV ± SD*	16.6±14.0	23.5±25.0	13.3±7.3	11.7±10.0
Middle	*Mean CV ± SD*	13.5±12.8	19.1±21.0	9.5±7.6	12.6±8.9
Late	*Mean CV ± SD*	15.3±15.7	17.8±11.6	11.2±12.5	10.7±7.8
Plateau MVC	*Mean CV ± SD*	9.0±7.2	18.0±12.2	12.6±11.4	10.7±6.0
Normalised MEP amplitude		*VM*	*VL*	*RF*	*Whole Quad*
Early	*Mean CV ± SD*	18.0±14.7	28.9±22.2	14.2±9.2	14.0±9.8
Middle	*Mean CV ± SD*	13.8±11.6	23.3±15.1	14.6±9.1	10.0±7.8
Late	*Mean CV ± SD*	17.3±15.0	21.5±16.7	13.4±11.0	11.0±5.1
Plateau MVC	*Mean CV ± SD*	12.7±11	23.1±14.1	12.4±12.2	10.0±7.2
Silent Period		*VM*	*VL*	*RF*	*Whole Quad*
Early	*Mean CV ± SD*	7.8±8.4	7.9±8.4	8.9±8.7	9.7±9.2
Middle	*Mean CV ± SD*	8.4±7.8	9.0±7.6	8.2±6.9	7.9±7.1
Late	*Mean CV ± SD*	8.3±5.2	7.7±5.6	8.0±4.7	7.8±5.7
Plateau MVC	*Mean CV ± SD*	4.0±3.9	5.0±3.3	3.7±3.1	4.1±3.4
